# Validity in the Era of Competency-Based Health Professions Education: Tensions and Potential Solutions

**DOI:** 10.5334/pme.2593

**Published:** 2026-09-29

**Authors:** Michael S. Ryan, Claire Touchie, Jason R. Frank, H. Carrie Chen, Benjamin Kinnear, As members of the International Competency-Based Health Professions Education Collaborators

**Affiliations:** 1 Department of Pediatrics, University of Virginia School of Medicine, Charlottesville, Virginia, United States of America; 2 Centre for Innovation in Medical Education, University of Ottawa, Ottawa, Ontario, Canada; 3 University of Ottawa, Canada; 4 The Ottawa Hospital, Canada; 5 Department of Health Systems Science, Kaiser Permanente Bernard J. Tyson School of Medicine, Pasadena, California, United States of America; 6 Department of Pediatrics, University of Cincinnati College of Medicine, Cincinnati, Ohio, United States of America

## Abstract

Assessment in health professions education has evolved dramatically in the past hundred years. The way in which we conceptualize validity has also required evolution, particularly in the era of competency-based health professions education (CBHPE). The CBHPE approach compels us to reexamine a historical focus on objectivity, individual assessments, and standards of achievement alone. In this manuscript, the authors explore the implications CBHPE places on validity, highlighting three tension points: the paradigm of assessment, levels of validation, and the reference for competence. The authors examine health profession education’s historical orientation toward the post-positivist paradigm including an emphasis on standardization, reliability, and truth-seeking. This is contrasted with two alternative paradigms, constructivism and pragmatism, which prioritize different data sources to build validity arguments. In the section on levels of validation, the authors explore tensions between validity arguments that are holistic (considers the entire picture of performance) and those which use a decomposition approach (distilling competence into individual components). Finally, the authors consider the value in measuring learner competence using norm-referenced standards, criterion-reference approaches, or the measurement of growth throughout (and beyond) their training. Ultimately, the authors propose a balanced approach to address each tension, considering the strengths and weaknesses of traditional methods with the modern needs of CBHPE.

## Introduction

Over the past hundred years, health professions education (HPE) assessment has evolved. During the measurement era, high-stakes multiple choice question examinations and standardization ruled the assessment landscape [1]. However, as assessment experts recognized the value of human judgement, other approaches (e.g., workplace-based assessment (WBA), simulation, etc.) were gradually embraced, and widely deployed [2,3,4]. More recently, assessment has been conceptualized through a systems lens, with multiple methods being integrated deliberately into a single program [5]. Measurement is not extinct, nor is judgment. However, these modern considerations, from more diversity in assessment portfolios to a focus on programmatic assessment [6,7]; have introduced additional complexity, uncertainty, and methodological diversity.

In addition to changes in assessment, thinking about assessment validity has also evolved in HPE. Building from the work of scholars in other fields [8,9], the conceptualization of validity as an argument about the interpretations and uses of assessment data has grown in use [10]. The argument-based approach provides flexibility in what and how much evidence we seek, while also allowing for methodologically diverse approaches to seeking such evidence [11]. Though the notion of validity as an argument remains prevalent [12] the shift toward competency-based health professions education (CBHPE) requires an examination of *how* validity arguments are developed. Instead of making arguments aimed only at single assessments, CBHPE endorses the integration of assessment systems and the measurement of growth and competency attainment to inform continued development during training. Validity arguments must now account for complex decision-making, such as claims of competence (or incompetence) made by a group of experts (i.e., a competence committee). Thus, CBHPE represents a shift in HPE’s perspective on validity.

In this manuscript, we explore three tensions associated with validity arguments in CBHPE: paradigms of assessment, level of validation, and reference for competence. By examining these tensions, we aim to elaborate on the challenges CBHPE presents to current conceptualizations of validity and, when possible, provide potential solutions to manage these tensions. A summary of the tensions is illustrated in Figure 1.

**Figure 1:**
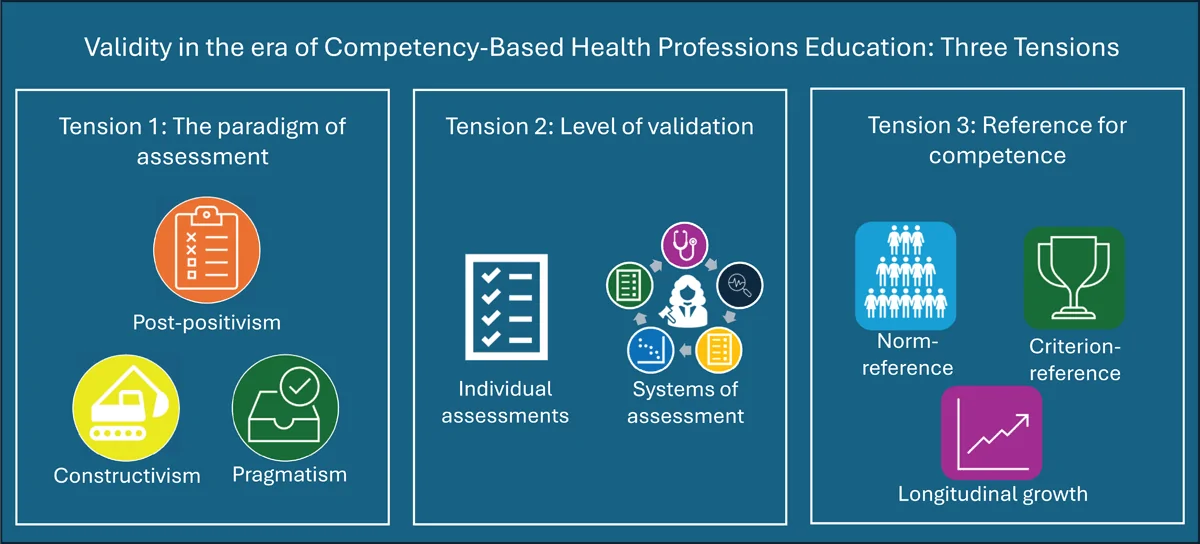
Inherent tensions to validity in the era of competency-based health professions education.

## Tension #1: The paradigm of assessment

In this section, we explore the impact of one’s paradigmatic orientation on the conceptualization of validity. We define the term *paradigm* as an *epistemological stance*, meaning the belief system (e.g., the nature of knowledge, the best approach to measure knowledge, etc.) that influences one’s perspective [13]. Herein, we focus on three orientations with relevance to CBHPE: post-positivism, constructivism, and pragmatism, though other paradigms exist within HPE.

### Validity evidence in a post-positivist paradigm

Since the mid to late 1800s, the post-positivist paradigm has dominated HPE assessment [1,14]. Much of the emphasis on a post-positivist approach arose from concerns over the competence of health care professionals. In response, Western cultures increasingly adopted official assessment boards and accrediting bodies, focusing efforts on high-stakes licensure and/or certification examinations, objectivity, standardization, fairness, and reliability to increase accountability to the public [15,16]. This *psychometric era* argued that subjectivity was undesirable, that real-world observations of performance were unfair, and that good assessments were those that were standardized and thus free from bias [4]. Using classical test theory as a foundation, the implicit assumption in this paradigm is that there is, in fact, a “true score” that reflects a learner’s consistent ability for a given domain. The goal is therefore to determine how much error (i.e., noise) is present in assessment data [17].

### Validity evidence in a constructivist paradigm

An alternative to the post-positivist paradigm involves a constructivist/subjectivist orientation to validity. Such an approach emerged over the past few decades as CBHPE experts highlighted the inherent ‘shared subjectivity’ [18] of competency decisions and the lack of standardization in workplace-based measurement [19]. Consequently, constructivists argue that we focus validity around sense-making, context, and social processes common to a subjectivist/constructivist orientation [4,19]. Rather than consider individual rater reliability, we should examine the reliability of a competence or readiness decision at the level of a competency committee (CC) [20], a group of individuals who use aggregate assessment data to render a summative determination [21]. Though quantitative methods may be useful in some circumstances (i.e., to measure the reliability of CC decisions across committees), evidence may incorporate more qualitative components in this constructivist paradigm.

### Validity evidence in a pragmatic paradigm

Finally, we consider a pragmatic orientation to validity. As Morgan summarizes, pragmatism is primarily concerned with “workability” and emphasizes shared meaning-making, mutual understanding, and collaboration among parties [13]. In the context of validity and competency-decision-making, this means that one prioritizes consequences evidence: the impact (benefits and harms) of the assessment and the decisions that follow [22]. Validity evidence in this paradigm can include the quality of the group decision-making process [23], and the reliability of decision-making *between* committees. In contrast to post-positivism or constructivism, pragmatism represents a more integrative (i.e., use of both quantitative and qualitative measures) [13] orientation to validity.

### The challenges in each paradigm

Though the pursuit of objectivity [24] has been widespread for more than a century, concerns over a post-positivist approach have been expressed for almost as long [4,24,25]. As Valentine and colleagues eloquently state, “[objectivity in assessment] can lead to naive trust in linear causality,” a fallacy that distills the assessment of complex professional tasks into that which can be measured, even when such measurement is not possible [24]. From a theoretical lens, authors have argued that all measurement is influenced by biases and that, therefore, objectivity is not attainable and may intentionally or unintentionally promote equity concerns [4,26]. Further, assessment in the workplace is inherently subjective and unstandardized, since it depends on perceptions of a human rater and the performance of the trainee at that time, in that context [17]. Finally, as Gingerich and colleagues share, assessors can be “meaningfully idiosyncratic,” [27] meaning that each assessor may only see one view and the combination of views gives us the truest picture of the learner [27]. Therefore, one should anticipate some degree of inconsistency (i.e., low reliability) in WBA due to authentic, and even meaningful, differences in perception, experience, or context [4,24,27].

However, there are challenges with constructivist and pragmatic paradigms as well. Competence is a social construct; and, as such, how one defines competence is inherently dependent on customs, norms, and social expectations [28,29]. Though this may be expected, it raises the question of whether notions of reliability at the level of committees are even attainable or merely a theoretically appealing concept [30]. There are also inherent risks to these paradigms that must be carefully managed. For example, evidence from CC decision-making literature suggests that many CCs default to anecdotes, lack formal training, and rely on norm-referencing, often ignoring or misunderstanding the intent of competency-based frameworks when rendering their decisions [31,32,33]. Consequently, it is likely that some inconsistencies among raters are not necessarily due to true differences in perception, performance, or context; rather, they are due to legitimate threats to validity and reliability (i.e., poor evidence of response-process validity) [34]. If not carefully mitigated, well-intentioned subjective, group-based decision-making can become arbitrary, biased, and anecdotal decision-making.

### Managing the tension

For the reasons outlined above, the tension in paradigmatic orientations in the CBHPE era is challenging to resolve. How does one effectively negotiate between useful subjectivity (i.e., shared subjectivity of experts) and arbitrary or anecdotal decision-making? How do we navigate the inherent subjectivity of competency assessment without compromising a dedication toward a more equitable system of assessment? How does one interrogate the reliability of committee ratings when competence is context (and perhaps committee) dependent? Does reliability at the CC level obviate the need for reliable data at the individual assessment level?

As Tavares et al. wrote [35], compatibility should be sought between paradigm, assessment, and validity. Rather than arguing for one paradigm over another, we suggest that the tensions may be managed through careful, considered alignment between paradigms and data sources (Table 1). The post-positivist paradigm is valuable in situations where standardization and objectivity are paramount [14]. This includes multiple-choice tests (e.g. certification examinations) and clinical assessments where standardization is sought, such as objective structured clinical examinations. In these situations, traditional psychometrics (e.g., item-response theory, inter-rater reliability) remain highly valuable. Similarly, constructivism is instrumental, particularly when an assessment involves individual judgment. A prime example involves WBAs, which are inherently dependent on raters and their context [19]. Importantly, psychometrics can still play a role in a constructivist paradigm. For example, generalizability theory can differentiate among sources of variance, thereby determining the extent to which score variation is attributable to raters, learners, or other factors [36,37]. Critically, generalizability theory cannot further delineate the source of rater differences (i.e., meaningful idiosyncrasies [27] versus construct-irrelevant influences), [38] and thus, subjective decision-making is still integral to workplace-based judgments. Finally, the pragmatic paradigm is invaluable for considering higher-order inferences related to group decision-making, as required for competence committee determinations. Effective group process is a framework for making such decisions [23] and a method that resonates with the pragmatic paradigm.

**Table 1:** Paradigms of assessment and application to validity in competency-based health professions education.


PARADIGMATIC ORIENTATION	DEFINITION	TYPES OF DATA FAVORED	EXAMPLES OF EVIDENCE	USE IN COMPETENCY-BASED HEALTH PROFESSIONS EDUCATION

Post-positivism	There is a “true score” that reflects a learner’s ability in a specific competency domain	Objective, numerical, standardized data	Classical test theory Item-response theory Inter-rater reliability	Standardized test settings (e.g. multiple-choice item tests, objective structured clinical examinations, etc.)

Constructivism	There is no “true score,” the definition of competence is socially constructed	Subjective, qualitative, non-standardized data	Thematic analysis Generalizability theory*	Workplace-based assessments

Pragmatism	Competence is developed through a mutual understanding and “workability” between groups	Supports a combination of qualitative and quantitative data	Effective group process	Group decision-making (i.e. competency committees)


*Generalizability theory can offer sources of variance (i.e. raters vs. learners) but cannot provide a more specific delineation within the source (i.e. meaningful rater differences vs. construct-irrelevant variance).

In summary, the notion that one can distill a complex social field into purely numerical and quantifiable measurements is unrealistic and theoretically unfounded [4,18]. We would therefore advocate against exclusive reliance on standardized tests to support advancement, competency decisions, or residency selection, despite demands to the contrary [39,40]. Similarly, though, we acknowledge that the reliance on purely subjective decision-making is problematic due to risk of numerous biases [41] and inadequate shared mental models [31]. For these reasons, we recommend a balanced approach that fosters alignment among the purpose of assessment, paradigmatic orientation, and the collection of validity evidence.

## Tension #2: Level of validation

Our second tension concerns whether we focus our validity argument on individual components of the assessment program or consider it more holistically. To begin this discussion, we consider the origin of modern validity practices and the focus on evidence for individual instruments.

### Modern validity theory and the ‘validation’ of individual instruments

In conjunction with the post-positivist focus of assessment culture in the 20th century, HPE programs frequently interrogated the validity of assessments at the level of individual instruments [8,9] Each instrument was evaluated to determine the relative sufficiency of the evidence to support (or refute) the interpretation of the findings. The higher the stakes, the more evidence was required [38]. Though commonly used frameworks allowed for comparisons between instruments (e.g., Messick’s *relations to other variable evidence* [8], or Kane’s *extrapolation*) [42], such comparisons were designed to test the instrument in question against other similar or dissimilar measures.

When applied to high-stakes quantitative assessments (e.g., certification examinations), such an approach is logical. Since the goal is to judge a trainee at a particular moment in time, the validity of a single assessment is paramount. However, in recent decades, the number and diversity of assessments used in HPE programs have grown. Moreover, experts in assessment and CBHPE have advocated considering not only individual assessments but also complex systems of assessment [5]. In CBHPE, it is thus not only imperative to understand the validity of individual assessments but also the validity of the entire assessment program. This has necessitated a new approach to assessment practices, with an impact on our validation practices.

#### The importance of a programmatic assessment approach

Programmatic assessment is defined as the intentional collection of a wide variety of data from multiple data sources across different contexts for formative purposes, which is then collated for later summative decisions [1]. Examples of data commonly collected include WBAs, objective structured clinical examination scores, and written examination scores. More recently, educational leaders have pioneered the use of clinical data, such as data originating from the electronic health record and from ambient artificial intelligence [49,50,51].

These various assessments are assembled into an assessment system that functions as an integrated ecosystem of instruments and processes, with decisions occurring at multiple levels [5]. In programmatic assessment, such frontline data are aggregated and synthesized for review by a competency committee to make higher-stakes decisions about readiness for progression through training or graduation/credentialing [6,7]. In theory, a group process for synthesizing assessment data from multiple parts of the system allows for the most well-rounded and complete picture of a learner’s performance and, thus, clarity about their progress toward competence.

#### Obtaining validity at a systems level: holistic decisions or decomposition

The multifaceted systems of assessment used in CBHPE create a critical question: How do we develop a validity argument for an entire system of assessment? Should we consider competence as a pure holistic judgment, or should we consider the final judgment additive and based on the addition of various data points from (individually) validated assessments?

In educational settings outside of medical education, this is often referred to as a tension between decomposition (i.e., decomposing competence into measurable units) and holism (i.e., viewing competence as a holistic judgment) [43]. The decomposition view seeks to make competency determinations by summing individual units together. The assumption is that if individual components are valid, the final summative decision will be valid as well. A holistic view focuses more explicitly on the overall decision. The assumption is: the addition of individual components will be insufficient to capture the complexity of an overall competency judgment, and thus, a holistic determination is required [43].

#### Can we navigate the tension?

This tension is not simple, since the debate between decomposition and holism can play out at all levels. Individual assessment instruments can be holistic (e.g., entrustment-supervision ratings) or decomposed into units (e.g., subcompetency ratings), as can competency committee decisions. Moreover, multiple assessments can speak to a competency, and each assessment can measure more than one competency [44,45]. In this way, validity may be better viewed as the degree to which pieces of evidence speak to achievement of that one competency.

One potential strategy to navigate this tension involves taking a both/and rather than an either/or approach by recognizing the benefits of considering the larger assessment system *and* collecting validity evidence for individual assessments. A useful analogy may be the construction of a building. Evidence that the parts (steel, rivets, concrete) are high-quality and reliable would ensure the building is made of solid materials. However, evidence is also needed that the engineering decisions were appropriate, the pieces fit together properly, and the resulting building is structurally sound.

Similarly, for each assessment, validity evidence could be collected to support the resulting data. Using WBAs as an example, evidence could be collected to establish that raters truly observe what they are rating and understand the assessment task (response process evidence). Other evidence could include whether important skills are assessed during observations (content evidence) or whether the data collected provide useful feedback to inform learner growth (consequences evidence) [8]. However, evidence of validity for the downstream competency committee decisions might also be sought [20]. Evidence showing that sufficient numbers of observations were made to inform higher-stakes decisions (internal structure) or that CC members understand decision-making processes and the assessment data they interpret (response process) would be meaningful for the validity argument supporting the assessment system as a whole. Throughout this process, it is critical to view assessments through a justice-oriented lens [46,47], with extreme care to ensure that all learners, particularly those from minoritized backgrounds, are not disadvantaged by programs of assessment created for/by the dominant physician archetype.

Collecting validity evidence supporting all levels within a system of assessment, and for the system itself, is a resource-intensive endeavor. Thus, the tension is perhaps more pragmatic than theoretical: do educators and programs have the capacity to collect validity evidence at all levels and then connect the evidence into a coherent argument for ultimate decisions? In some settings, the resources required may be prohibitive, both from a cost and personnel standpoint. And further, if we adopt a programmatic assessment approach, how do we navigate the dual-purpose assessments serve (both a formative assessment for learning and a summative assessment for decision-making) [48]?

Due to these concerns, the opportunity to collect validity evidence must therefore be viewed as a longitudinal, iterative, and collaborative process that can be its own program of scholarship. Educators can prioritize areas that are most important or under the most scrutiny in early efforts. For example, if WBAs make up the largest part of an assessment system, validation efforts might start there. Similarly, if there are concerns about a competency committee’s processes in synthesizing assessment data into summative decisions, then that may be the best place to focus early efforts. Finally, assessment experts can prioritize which assessments truly require validation in their context and which instruments have sufficient evidence of validity from similar contexts.

The overall validity argument can then fit together like the building in the construction analogy. As component pieces of the system are supported by validity evidence, the system’s overall structure is strengthened. As the building takes shape, a validity argument can be made for the final form. Without evidence of validity supporting the component parts of an assessment system, the data informing program-level decisions may be questionable. Conversely, if validation focuses only on individual instruments, the program as a whole may lack evidence of cohesion and defensibility, particularly if attention is not given to the alignment between assessments and the program-level decision. Educators face a tension between ‘zooming in’ (i.e., decomposition) on component parts and ‘zooming out’ (i.e., holistic determination) to the entire assessment program when building validity arguments [43]. We also suggest that educators consider strategies to manage the dual purpose of assessments within the CBHPE paradigm; several helpful suggestions are provided in an article by Ryan and colleagues, which is also included in this supplement [48].

## Tension #3: Reference for Competence

The final tension we consider concerns the standard we use to determine competence. A defined level of competency attainment is necessary to determine readiness for progression in training or credentialing such as completion of training, certification, or licensure. Clear outcomes standards are rooted in social accountability [49], and are necessary to ensure that all trainees have met a given standard. There are three general approaches one may consider: norm-reference, criterion-reference, or longitudinal growth. In this section, we explore each of those approaches.

### A norm-reference standard

In traditional approaches to validity, differences in achievement between individuals (i.e., between-subject variance) are commonly used to establish thresholds for competence [50]. The notion of between-subject variance is foundational to traditional validity arguments; measurement of reliability (e.g., Cronbach’s alpha, generalizability theory) relies on individual variance, with the requirement that individual achievement must vary from one person to the next [17,50] Such an approach is inherently norm-referenced; it assesses attainment using comparative measures within a population.

### A criterion-reference standard

The requirement for between-subject variance is contrary to the underlying premise of CBHPE. By definition, CBHPE is an outcomes-based approach to education and training [51], and, as such, the paradigm is concerned with *when*, not *if*, a learner achieves a threshold of competence to demonstrate accountability to the public. Moreover, variance, particularly in the clinical environment, may be low. The plateau effect describes this phenomenon, in which all learners meet the defined outcome and, thus, differences among learners are negligible [52]. In these situations, determining the threshold for competence using between-subject variance alone runs the risk of defaulting to norm-referencing and bias, making a decision about one’s competency by comparing the trainee to peers rather than to established criteria [53]. Therefore, CBHPE proponents argue for criterion-based approaches [53], many of which are now commonplace for many high-stakes examinations. Examples include standard-setting procedures, which use expert consensus combined with assessment data to establish an acceptable achievement threshold [54].

### A longitudinal growth standard

An appealing third option is within-subject variance, or, in other words, the growth of the healthcare professional throughout training *and* throughout their careers. Healthcare professionals acquire skills at different rates; thus, monitoring growth appears feasible and theoretically sound through both an equity- and competency-based lens [52,55,56]. We may measure an individual’s growth or learning toward a defined outcome and offer targeted interventions along the way. For example, Park and colleagues recently described growth curves among family medicine residents, identifying patterns predictive of meeting competence thresholds and those requiring remediation [57]. Importantly, growth is not restricted to formal training; a hallmark of healthcare professions is a commitment to lifelong learning to ensure the maintenance of competence and, where appropriate, the acquisition of new competencies [58].

### How can we navigate the tension?

There are significant conceptual arguments against norm-referenced approaches to competency attainment [51,53]. However, many criterion-based approaches (e.g., Hofstee and Angoff methods) [59] still include some degree of learner-level data to generate criteria and are, therefore, still prone to norm-referencing [60]. Focusing efforts on growth rather than attainment alone sounds appealing and relatively simple. However, is there an expected growth trajectory? If so, what deviation from that is within the range of typical development versus outside this expected range? How does one know that individual growth reflects true improvement versus construct-irrelevant variance? How does one interpret a learner with a flat growth curve who has achieved a predetermined level of competency? How do we account for loss of competency over time?

Bok et al. [61] have demonstrated that individual growth can be measured over time using random coefficient modeling, with three different assessment tools in their programmatic assessment strategy for veterinary students, using a competency-based approach. In other studies, longitudinal learning trajectories were modeled using latent class growth curve models, revealing patterns of upward growth [57,62]. These studies demonstrate that measurement of growth is possible, though admittedly more complex.

However, the measurement of growth must be balanced against our accountability to the public; growth is important, but only to the extent that the learner ultimately meets the threshold for competent practice. For these reasons, it is likely necessary to both define minimum competency standards and measure progress toward those standards. Such balance between norm- and criterion-referenced standards, in addition to longitudinal growth tracking, ensures feedback and remediation to learners who are struggling so all meet the minimum standards for promotion, graduation, or credentialing.

## Conclusions

Assessment in the health professions is undergoing a fundamental change. The CBHPE movement represents a foundational shift in educational philosophy across healthcare programs. This change represents not only a reform in program objectives and curricular content, but also a reconsideration of how we compose validity arguments based on assessment data. There are at least three major points of tension as we reconsider validity in the era of CBHPE. Though these tension points represent a clash of philosophies, they also present an opportunity to strike a balance between old and new approaches, thus promoting a deeper realization of CBHPE. Moving forward, 21st-century assessment of health professionals should be programmatic, designed to construct a validity argument, and take into account tensions related to authentic WBA, input from multiple types of assessments, and a developmental view of competence.
